# Critical dynamics predicts cognitive performance and provides a common framework for heterogeneous mechanisms impacting cognition

**DOI:** 10.1073/pnas.2417117122

**Published:** 2025-04-03

**Authors:** Paul Manuel Müller, Gadi Miron, Martin Holtkamp, Christian Meisel

**Affiliations:** ^a^Computational Neurology, Department of Neurology, Charité—Universitätsmedizin Berlin, Berlin 10117, Germany; ^b^Computational Neurology, Berlin Institute of Health, Berlin 10178, Germany; ^c^NeuroCure Cluster of Excellence Charité—Universitätsmedizin Berlin, Berlin 10117, Germany; ^d^Epilepsy-Center Berlin-Brandenburg, Institute for Diagnostics of Epilepsy, Berlin 10365, Germany; ^e^Epilepsy-Center Berlin-Brandenburg, Department of Neurology, Charité—Universitätsmedizin Berlin, Berlin 10117, Germany; ^f^Bernstein Center for Computational Neuroscience, Berlin 10099, Germany; ^g^Center for Stroke Research Berlin, Berlin 10117, Germany

**Keywords:** network dynamics, epilepsy, cognition

## Abstract

The brain criticality hypothesis postulates that brain dynamics are set at a phase transition where information processing is optimized. Long-range temporal correlations (TCs) characterizing the dissipation of information within a signal have been shown to be a hallmark of brain criticality. However, the experimental link between cognitive performance, criticality, and thus TCs has remained elusive due to limitations in recording length and spatial and temporal resolution. In this study, we investigate multiday invasive EEG recordings of 104 persons with epilepsy (PwE) together with an extensive cognitive test battery. We show that short TCs predict cognitive impairment. Further, we show that heterogeneous factors, including interictal epileptiform discharges (IEDs), antiseizure medications (ASMs), and intermittent periods with slow-wave activity (SWSs), all act directly to perturb critical dynamics and thus cognition. Our work suggests critical dynamics to be the setpoint to measure optimal network function, thereby providing a unifying framework for the heterogeneous mechanisms impacting cognition in conditions like epilepsy.

## Significance

Cognitive function emerges from cortical network dynamics and is often impaired in neuropsychiatric disorders like epilepsy. Physics and information theory suggest that brain networks operate optimally at a critical state between order and disorder. Using comprehensive cognitive testing and multiday intracranial EEG from persons with epilepsy (PwE), we demonstrate that proximity to critical dynamics predicts cognitive performance across multiple domains. We show that heterogeneous factors known to impact cognition—including interictal epileptiform activity, antiseizure medications (ASMs), and sleep-like episodes—all act on one common endpoint: to perturb the critical state. Together, these findings support the concept of criticality as a unifying framework linking cortical dynamics and function and for understanding how different mechanisms affect cognition in neurological conditions.

Cognitive function is an emergent property of cortical network structure and dynamics and is often impaired by heterogeneous mechanisms in neuropsychiatric disorders (1–3). In persons with epilepsy (PwE), cognitive impairment is a common comorbidity severely impacting daily functioning and quality of life (4). PwE demonstrate a large heterogeneity in their cognitive function, with up to 70% of individuals having impaired cognition, most commonly in the language, memory, and executive domains (5). The causes of cognitive dysfunction in epilepsy are multifactorial, stemming from both the underlying etiology of the disease, such as mesial temporal sclerosis, as well as dynamic factors including effects of antiseizure medications (ASMs), interictal and ictal activity, and disrupted sleep patterns (6, 7). Some of these factors, including interictal spikes, are known to also contribute to cognitive dysfunction in other diseases like Alzheimer’s (8–10). Despite established associations of these factors, the neuronal mechanisms underlying cognitive function, and the differential impairment of cognitive domains remain largely not understood.

Central to intact cognitive function is the brain’s ability to process and integrate information across spatial and temporal domains (11–14). Physics and information theory have provided a framework describing an optimal state of information processing. This critical state, poised at the phase transition between chaotic and ceasing neuronal activity, is characterized by an equilibrium between excitation and inhibition in the neuronal network (15–17). When a network of neurons operates near a critical phase transition point, a range of information processing functions, including information transmission, integration, storage, dynamic range, and sensitivity to inputs, are optimized simultaneously (18–23).

The relevance of criticality in cortical networks is supported by experimental observations from animals and humans showing power-law scaling (24, 25) and long-range temporal correlations (TCs) (21, 26) which are hallmarks of critical network dynamics. Recent work has demonstrated that these signatures are predictably perturbed when networks are moved away from the critical point, either by external factors, such as ASMs (27, 28), or internal influences, such as continued wake and sleep deprivation (27, 29–32), providing further support for the relevance of critical dynamics in cortical networks.

While criticality provides a precise framework linking network structure to dynamics, its central claim—that critical dynamics predicts optimal network and thus cognitive function in humans—remains an area of active investigation. Prior work using noninvasive recording modalities has demonstrated associations between critical dynamics and specific cognitive processes, including motor learning, attention, and executive function. Critical dynamics have also been proposed as potential biomarkers for cognitive changes in conditions like ADHD and Alzheimer’s disease (33–41). However, key questions remain about how critical dynamics relate to broader cognitive function and how different mechanisms might affect cognition through perturbation of critical dynamics (40, 42, 43).

Building on this foundation, we utilize two large-scale intracranial EEG (iEEG) datasets, combined with comprehensive cognitive testing across multiple domains to demonstrate that proximity to the critical point robustly predicts cognitive impairment profiles in epilepsy. We show that heterogeneous factors, including interictal epileptiform discharges (IEDs), ASMs, and intermittent periods with slow-wave activity (SWSs), all act directly to perturb critical dynamics and thus cognition. Our work suggests critical dynamics to be the setpoint to measure optimal network function, thereby providing a unifying framework for the heterogeneous mechanisms impacting cognition in conditions like epilepsy.

## Materials and Methods

### Participants.

We conducted a retrospective analysis of persons with drug-resistant focal epilepsy (PwE) that underwent presurgical evaluation by intracranial video-EEG monitoring (Table 1). During monitoring, patients remained in the hospital bed, engaging in daily activities within these constraints, including resting, sleeping, meals, etc. We included two independent cohorts. The first dataset was composed of 81 PwE (35 females, average age 32.3 ± 10.7 y) assessed at the Berlin–Brandenburg Epilepsy-Center (dataset 1). The second dataset included 23 PwE (12 female, average age 28.5 ± 13.2 y) from the epilepsy center at the University of Freiburg, available through the Epilepsiae database (dataset 2) (44).

**Table 1.** Patient characteristics

	Berlin-Brandenburg epilepsy-center (dataset 1)	Epilepsiae dataset (dataset 2)
Number of patients	81	23
Sex Female/Male	35/46	12/11
Age	32.3 ± 10.7	28.5 ± 13.2
Epilepsy duration (years)	18.1 ± 12.8	19.3 ± 12.5
History of focal to bilateral tonic clonic seizures	71	8
Total number of ASMs (previous and current)	5.9 ± 2.6	2.7 ± 1.3
MRI findings		
MTS	12	8
Nonlesional	28	5
Focal cortical dysplasia	10	7
Others (low grade tumor, vascular lesions, lesions of unclear significance)	31	3
Epilepsy localization		
Temporal lobe	47	19
Frontal lobe	29	3
Occipital/parietal lobe	5	1
Seizure onset side		
Left	48	10
Right	33	5
Bilateral	0	8
Surgical outcome		
Engel 1	50	14
Engel 2–4	24	6
Neuropsychological testing, impaired/not impaired		
Language	54/27	NA
Verbal memory	32/49	NA
Attention	17/64	NA
Working memory	37/44	NA

ASM = Antiseizure medication. MRI = Magnetic resonance imaging. FCD = Focal cortical dysplasia.

### Cognitive Testing.

Cognitive testing was performed in all 81 subjects in dataset 1. Specifically, 14 tests corresponding to four cognitive domains were performed during routine neuropsychological evaluation, on average 6.8 ± 4.1 mo prior to iEEG monitoring. Cognitive domains evaluated included language, attention, working/short-term memory, and verbal learning and memory. Test distributions and further details are provided in *SI Appendix*, *Supplementary Material 7*. Cognitive domain impairment was defined as two parameters within a domain having a score of at least one SD below the mean of the norm population.

### Quantification of ASMs.

ASMs were normalized by their defined daily dose and summed up for each day. For each patient that underwent ASM tapering as part of the clinical video-EEG monitoring, days with the highest and lowest summed ASM dose were identified (high and low ASM days). Days with rescue medication (midazolam, diazepam, or lorazepam) were excluded due to their strong neurophysiological effects. Subjects without medication tapering were excluded from analyses related to ASM effect.

### Preprocessing of Intracranial Electroencephalography Data.

In dataset 1, iEEG data were available for the first 5 min of each hour during the multiday monitoring period, whereas in dataset 2, continuous iEEG data from two days were included. IEEG was preprocessed as follows: notch-filtering removed power line noise at 50 and 100 Hz; signals were down-sampled to a common frequency of 256 Hz (original sampling frequencies varied between 256 to 2,048 Hz) by using an antialiasing filter and then decimating the signal; bandpass-filtering (0.1 to 128 Hz) removed slow drifts and high-frequency artifacts; channels with constant signal or abnormal frequency peaks (>6 times interquartile range across the recording) were removed; finally, visual inspection verified the preprocessing and identified additional clearly artifactual channels for removal. Seizure segments were excluded along with 10-min pre- and postictal segments, based on onset and offset times marked by clinical experts.

### TCs Calculation.

TCs have been shown to characterize information processing, and their maximization is a hallmark for criticality (29, 45, 46). Here, TCs are calculated as previously described (27, 29). In detail, high-*γ* power fluctuations are evaluated as they most closely capture local spike rate variations (47–49). Therefore, median high-*γ*-powers (56 to 96 Hz) are calculated in nonoverlapping 125 ms windows using Welch’s method (Hann window) providing 125 ms temporal and 8 Hz frequency resolution. Next, powers are normalized through applying the logarithm (base 10). Then, autocorrelation functions of the high *γ*-power time series are calculated in consecutive 120-s windows (90 s overlap) for each iEEG channel. TCs quantify the autocorrelation function decay, defined as the time when the autocorrelation first drops below half the difference between the first lag value and the baseline (median of 40 to 60 s autocorrelation). Thus, lag values span almost 3 orders of magnitude, i.e., 0.125 to 60 s. The autocorrelation function value at lag zero is excluded as it is one by definition and thus independent of inherent noise and data quality levels, and its inclusion may obscure the true underlying autocorrelation function decay rate. As a result, the minimal value for TCs is 125 ms. To ensure robustness of the TCs, autocorrelation functions are first aggregated (median) over multiple 120-s windows. In case of assessing global states, e.g., comparing low and high ASM days, we subsampled to the number of time points in the minority group. Subsampling was repeated 100 times, and results were averaged. TCs from time-shuffled high-*γ*-power series serve as surrogate controls, preserving power distributions while destroying temporal relationships.

### Detection of Slow Waves.

We classified periods with slow-wave activity (slow-wave sleep, SWS) across the full wake-sleep continuum. SWS was scored on 30-s windows using a validated algorithm (50). First, a vigilance index is calculated as the fraction of the powers

$$\frac{\theta +\delta }{\alpha +\beta_{high}+spindle}.$$

Second, SWS was defined as one SD over the mean for each day separately (50). In order to align these 30-s segments with the TC analysis based on 120-s segments, we chose to define a 120-s window to be marked as “SWS” if at least one of the four 30-s segments in it was identified as SWS. Otherwise, the 120-s segment was labeled “nonSWS.”

### Detection of IEDs.

IEDs were identified using an automated deep-learning method that has previously been described and validated (51). In short, a template matching filter identified possible IEDs activity within each channel. Then, to reduce the dimensionality, the raw EEG signal was transformed into spectrograms and pretrained deep neural network classified the segments as IEDs. To analyze the effect of IEDs on TCs, we compared TCs in segments and channels without IEDs and with (5–30) IEDs per minute. Channels with less than 50 segments in both categories were excluded to ensure sufficient sampling of the autocorrelation function.

### Spectral Power Calculation.

Spectral powers were used as control measures when evaluating the relationship between TCs and cognitive impairment. We calculated the median power for every 120-s segment in the following bins using Welch’s method (Hann window): *δ* (0.5-4 Hz), *θ* (4 to 8 Hz), *α* (8 to 12 Hz), *β* (12 to 30 Hz), *γ* (30 to 45 Hz), and high-*γ* (55 to 95 Hz).

### Statistical Analysis.

For analyses comparing states of the same patients (ASM, SWS, and between IED bins), we used paired Wilcoxon.

To compare significance across patients with respect to cognition, we applied Brunner–Munzel tests as distributions of TCs are nonnormal and heteroscedastic. We corrected for multiple comparisons using the Benjamini–Hochberg method at a corrected α=0.05. The effect size was reported as the nonparametric relative effect. Finally, *P*-value distributions were compared with Kolmogorov–Smirnov tests against uniform distributions, which one expects under the null hypothesis.

### Neural Network Modeling.

We investigated TCs in a parsimonious neural network model, whose distance to criticality can be tuned by one parameter, and which has built-in mechanisms modeling ASM effects, SWS, and IEDs, similar to the model in (27–29). The model consists of N=1024 neurons on a 2-dimensional equidistant grid with periodic boundary conditions. Neurons are all-to-all connected with random uniform strengths scaled by a distance-dependent Gaussian profile

$$e^{-\frac{r_{\mathrm{ij}}^{2}}{2\sigma^{2}}}.$$

The Euclidian distance between neuron *i* and *j* is $r_{\mathrm{ij}}$ and *σ* is the scaling width of the profile which was set to σ=4 as in (52, 53). We omitted self-connections and randomly set 20% of neurons to be inhibitory and 80% to be excitatory, resulting in the weight matrix *w*. The strength of the weights is scaled by a constant factor changing the largest absolute eigenvalue *λ* of the weight matrix likewise (*SI Appendix*, *Supplementary Material 1*). Therefore, we report *λ* as the control parameter characterizing the connectivity.

At time *t*, neuron *i* can be active $s_{i}\left(t\right)=1$ or inactive $s_{i}\left(t\right)=0$. At t+1, a neuron will become active with probability

$$p_{i}\left(t+1\right)=\left\{\begin{array}{c} 0\;\text{if}\;\sum_{j}w_{\mathrm{ij}}s_{j}(t)<0\\ 1\;\text{if}\sum_{j}w_{\mathrm{ij}}s_{j}(t)>1\\ \text{else}\sum_{j}w_{\mathrm{ij}}s_{j}(t) \end{array}.\right.$$

Additionally, one neuron is set to be active at each of the $\max_{t}=5,000$ time steps as background activity.

Following previous work, the influence of ASMs is modeled by reducing the outgoing excitatory connection strengths by a factor $f_{exc}$, motivated by the mechanism of ion-channel blockers (28). Deep slow-waves sleep (SWS) is represented by introducing off-periods at random times where all neurons remain quiescent with probability $p_{Off}$ (29). To model IEDs, we introduced local spikes by setting a random neuron and its local neighbors (20% of all neurons) to be active with probability $p_{IED}$. Model simulations are repeated 1,000 times. TCs were extracted from the autocorrelation function of the average neuron firing after 500 timesteps to account for initial transients.

### Ethics Statement.

This study was approved by the Institutional Review Board of Charité—Universitätsmedizin Berlin. Due to the retrospective nature of the study, informed consent of patients was waived. The use of patient data from the Epilepsiae database was approved by the Institutional Review Board of the University of Freiburg and written informed consent that the clinical data might be used and published for research purposes was given by all patients (44).

## Results

We first demonstrate that heterogeneous mechanisms known to act on cognition: ASMs, IEDs, and intermittent periods of SWS, all act to perturb the optimal critical network state as a common target. Second, we show that the proximity to this critical network state is the strongest predictor of cognitive performance, thus suggesting it to be the setpoint to monitor cognition.

### Disruption of Optimal Critical Network State by Heterogeneous Mechanisms in a Network Model.

**Fig. 1.**
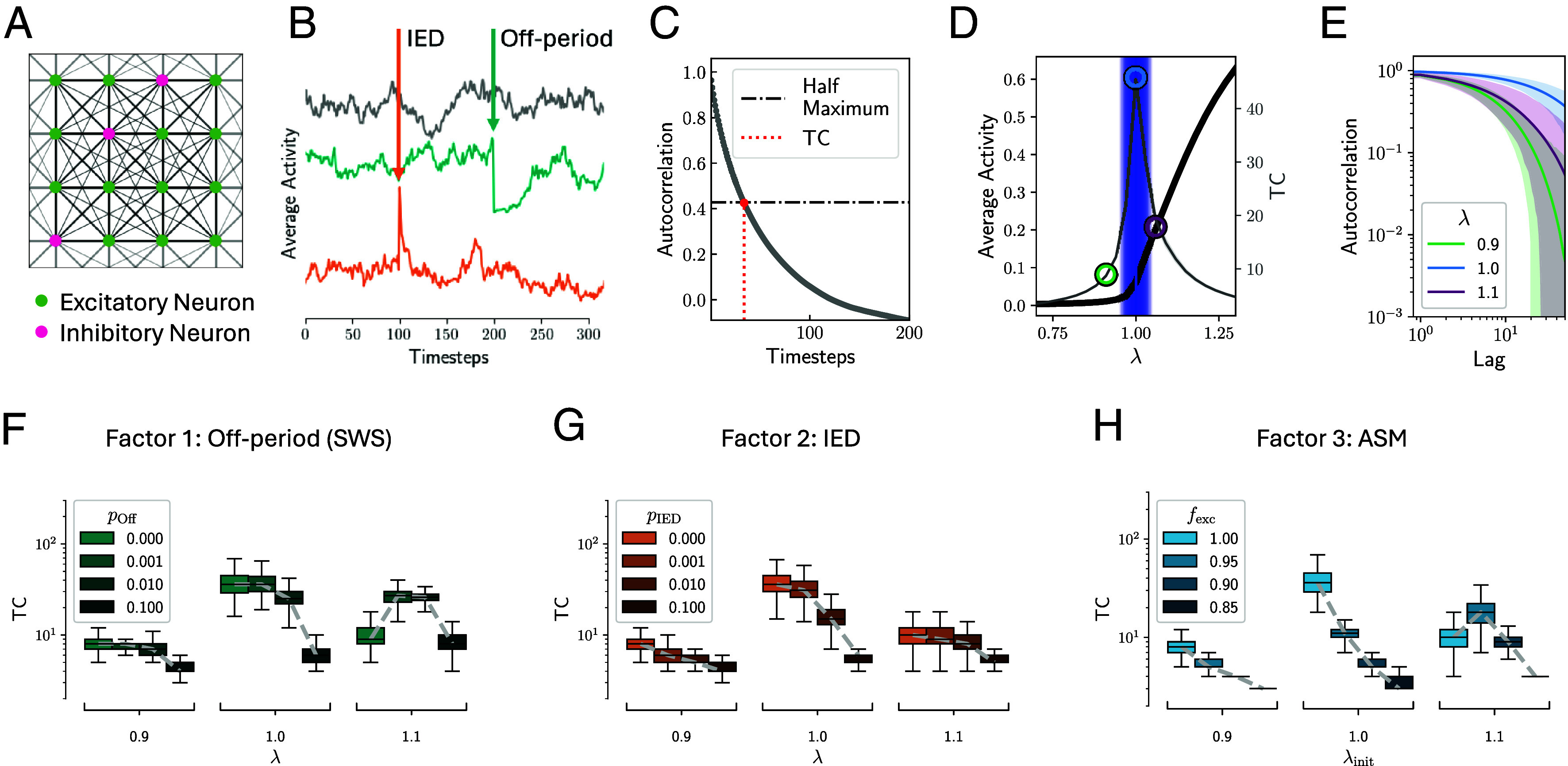
Neuronal network simulations indicate maximal TCs around the critical point, and their decline under SWSs, IEDs, and ASMs. (*A*) Networks of size N=1024 (20% inhibitory, 80% excitatory neurons) are coupled with distance dependence on a grid with period boundary conditions. (*B*) Unperturbed model run at criticality (gray), with an off-period (at timestep 200, teal), with an IED (at timestep 100, orange). (*C*) Definition of TC as the half width at half maximum of the autocorrelation function. (*D*) Phase transition of the order parameter (average activity, black) at which TCs (gray) peak. (*E*) The autocorrelation function for three different $\lambda \in \left\{0.9,1.0,1.1\right\}$ decays slowest for the critical λ≈1. (*F*) TCs as a function of SWS in the subcritical (λ=0.9), critical (λ=1), and supercritical (λ=1.1) regimes. (*G*) TCs as a function of IEDs in all dynamical regimes. (*H*) TCs as a function of ASMs.

We first analyze a parsimonious neuron network model based on a branching process with a well-established phase transition at a largest absolute eigenvalue of λ≈1 (17, 27, 54). *λ* characterizes how neural activity propagates through the network over time and can be influenced by factors like excitation–inhibition balance, neuronal excitability, and connection density between neurons (15). For λ≪1, the network was subcritical, i.e., the activity of the neurons ceased, and TCs were short (Fig. 1*D*). For λ≫1, the system was supercritical, i.e., the activity exploded, TCs remained short. At λ≈1 the system is critical which is supported by maximal duration of TCs (Fig. 1*D*). Fig. 1*E* displays the autocorrelation functions for λ∈{0.9,1.0,1.1}, showing that for λ=1, the autocorrelation function decays almost linearly in double-log coordinates, i.e., as a power-law, indicating that λ=1 is closer to criticality than λ=0.9 or λ=1.1. A perfect power-law cannot be expected due to the addition of background noise and the finite size of the model. Detrended fluctuation analysis is also commonly used to estimate TCs in time series. Consequently, in our model, TCs and Hurst exponents from detrended fluctuation analysis are tightly correlated and peak at criticality (*SI Appendix*, Fig. S3 *A* and *B*). Due to the finite model size and integration time, the phase transition is not sharp, i.e., activity does not saturate for all λ>1 (55). We next investigated the effects of SWSs, IEDs, and ASMs on TCs.

First, we modeled SWS as in ref. 29 (Fig. 1*B*). For the three dynamical regimes of the model, i.e., subcritical, critical, and supercritical, TCs declined under large $p_{Off}$ (Fig. 1*F*). Only in the supercritical regime, an increase of TCs for small $p_{Off}$ can be observed as effectively transients toward saturated dynamics are introduced. We observed the strongest decline in TCs if the system was initially close to the critical regime, λ=1.

Second, we introduced IEDs (Fig. 1*B*). For all model regimes, increasing $p_{IED}$ led to shortening of TCs, with the strongest effect seen again in the critical regime (Fig. 1*G*).

Third, we simulated the effect of ASMs (Fig. 1*H*). In the subcritical and critical regime, we found a reduction of TCs with decreasing $f_{exc}$. In the supercritical regime (λ=1.1), slight reductions of excitability lead to longer TCs, whereas larger decreases of $f_{exc}$ also lead to lower TCs.

In line with previous work, the model results indicated that optimal information processing should be found around a critical point where TCs are maximized. Conversely, SWSs, IEDs, and ASMs all shifted dynamics away from criticality, thereby reducing TCs as a potential signature of cognitive impairment.

### Disruption of Optimal Critical Network State by Heterogeneous Mechanisms in Human iEEG.

**Fig. 2.**
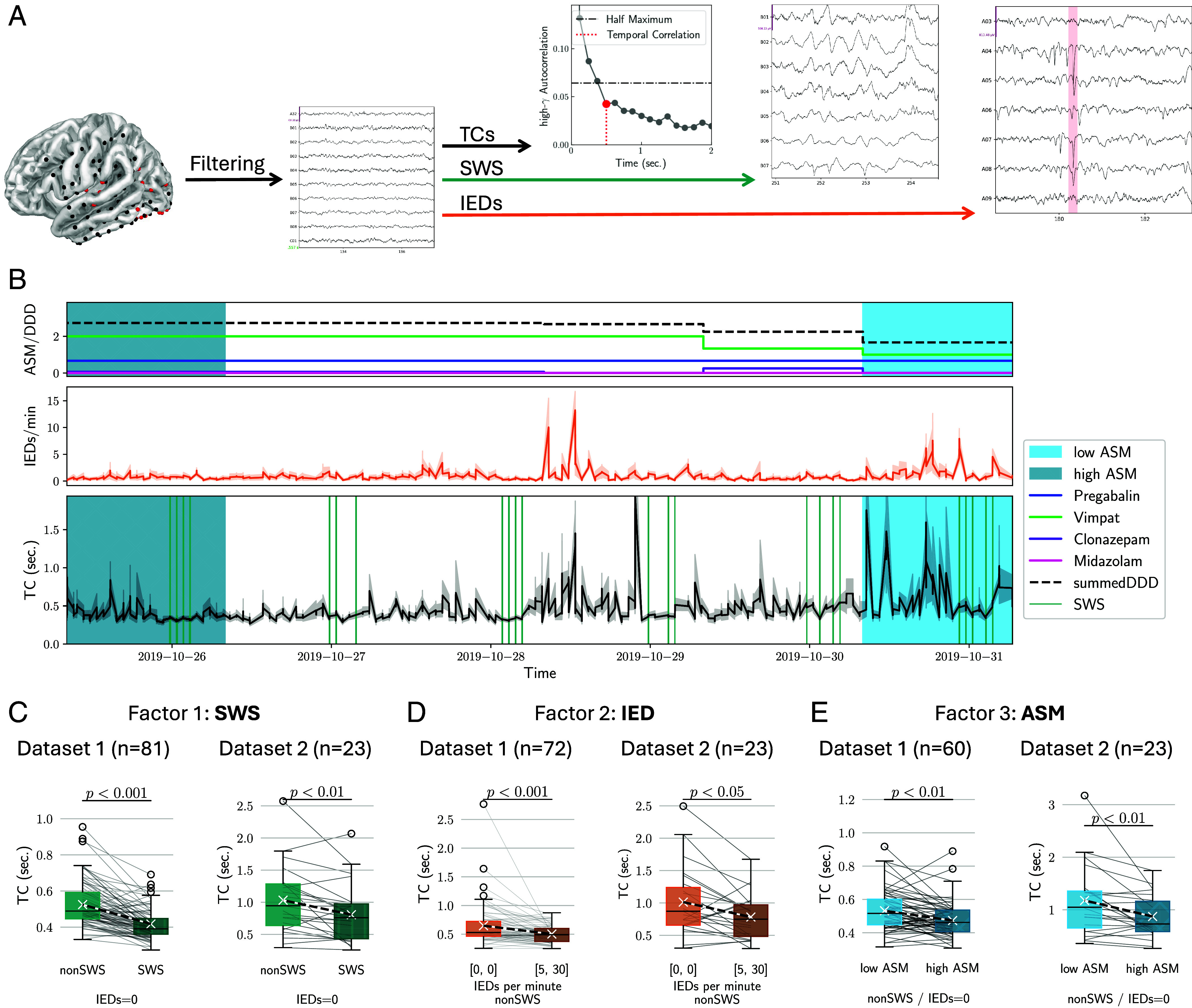
Disruption of TCs and network optimality through SWS, IEDs, and ASMs. (*A*) iEEG data from persons with epilepsy (PwE) followed by extraction of TCs and classification of segments with SWSs and IEDs. (*B*) For each patient, ASM dosage was recorded and aligned with IED rate, SWS segments, and TCs. (*C*) TCs as a function of SWS in the two datasets, excluding IED segments. (*D*) TCs as a function of IEDs, excluding SWS segments. E, TCs as a function of ASMs, excluding SWS and IED segments.

TCs from iEEG data of 104 PwE ranged between 0.125 and 5 s were correlated to Hurst exponents (*SI Appendix*, Fig. S3 *C* and *D*) and changed as a function of SWSs, IEDs, and ASM load (Fig. 2 *A* and *B*). Among the three factors, only SWS and IEDs demonstrated significant pairwise correlation (both datasets: P<0.001, Wilcoxon signed-rank test).

SWS epochs comprised (18±7)% of segments in dataset 1 and (19±6)% in dataset 2. TCs during SWS were significantly shorter compared to non-SWS segments (dataset 1: ${TC}_{nonSWS}=\left(0.53\pm 0.13\right)$ sec. vs ${TC}_{SWS}=\left(0.45\pm 0.08\right)$ sec., P<0.001; dataset 2: ${TC}_{nonSWS}=$ (1.0±0.5) sec. vs ${TC}_{SWS}=\left(0.8\pm 0.5\right)$ sec., P< 0.01; Fig. 2*C*). Segments with IEDs were excluded from the analysis due to the correlation of SWS with IEDs.

IED analysis revealed average rates of (2.6±2.4) IEDs/(channel ⋅ min) and (3.1±1.7) IEDs/(channel ⋅ min) in dataset 1 and dataset 2, respectively. TCs were longer in the absence of IEDs (dataset 1: ${TC}_{noIED}=\left(0.6\pm 0.4\right)$ sec. vs ${TC}_{\left[5,30\right]IEDs}=\left(0.5\pm 0.2\right)$ sec., P<0.001; dataset 2: ${TC}_{noIED}=\left(1.0\pm 0.5\right)$ sec. vs ${TC}_{\left[5,30\right]IEDs}=\left(0.8\pm 0.3\right)$ sec., P<0.05; Fig. 2*D*). SWS segments were excluded from the analysis due to the correlation between SWS and IEDs.

For ASM effects, we analyzed patients who underwent medication tapering (dataset 1: N = 60, 50 ± 30% reduction; dataset 2: N = 23, 70 ± 30% reduction). TCs were shorter under increased ASM loads (dataset 1: ${TC}_{lowASM}=\left(0.53\pm 0.12\right)$ sec. vs ${TC}_{highASM}=\left(0.48\pm 0.11\right)$ sec., P<0.01; dataset 2: ${\text{TC}}_{\text{low}\text{ASM}}=(1.2\pm 0.7)$ sec. vs ${TC}_{highASM}=\left(0.9\pm 0.4\right)$ sec., P<0.01; Fig. 2*E*). For this analysis, we excluded SWS and IEDs as potential confounders.

These effects were not observed in surrogate TCs. Furthermore, neither the raw high-*γ*-power nor the signal-to-noise ratio, estimated from the high-*γ*-oscillatory component, could explain the TC changes (*SI Appendix*, Figs. S2 and S6). While other commonly used measures, i.e., Hurst exponents and *α*–band TCs showed similar ASM-related trends, they were generally less robust than the high-*γ*-TCs (*SI Appendix*, Figs. S3 and S4, respectively).

### Optimal Network State Disruption Inside and Outside the Seizure Onset Zone.

**Fig. 3.**
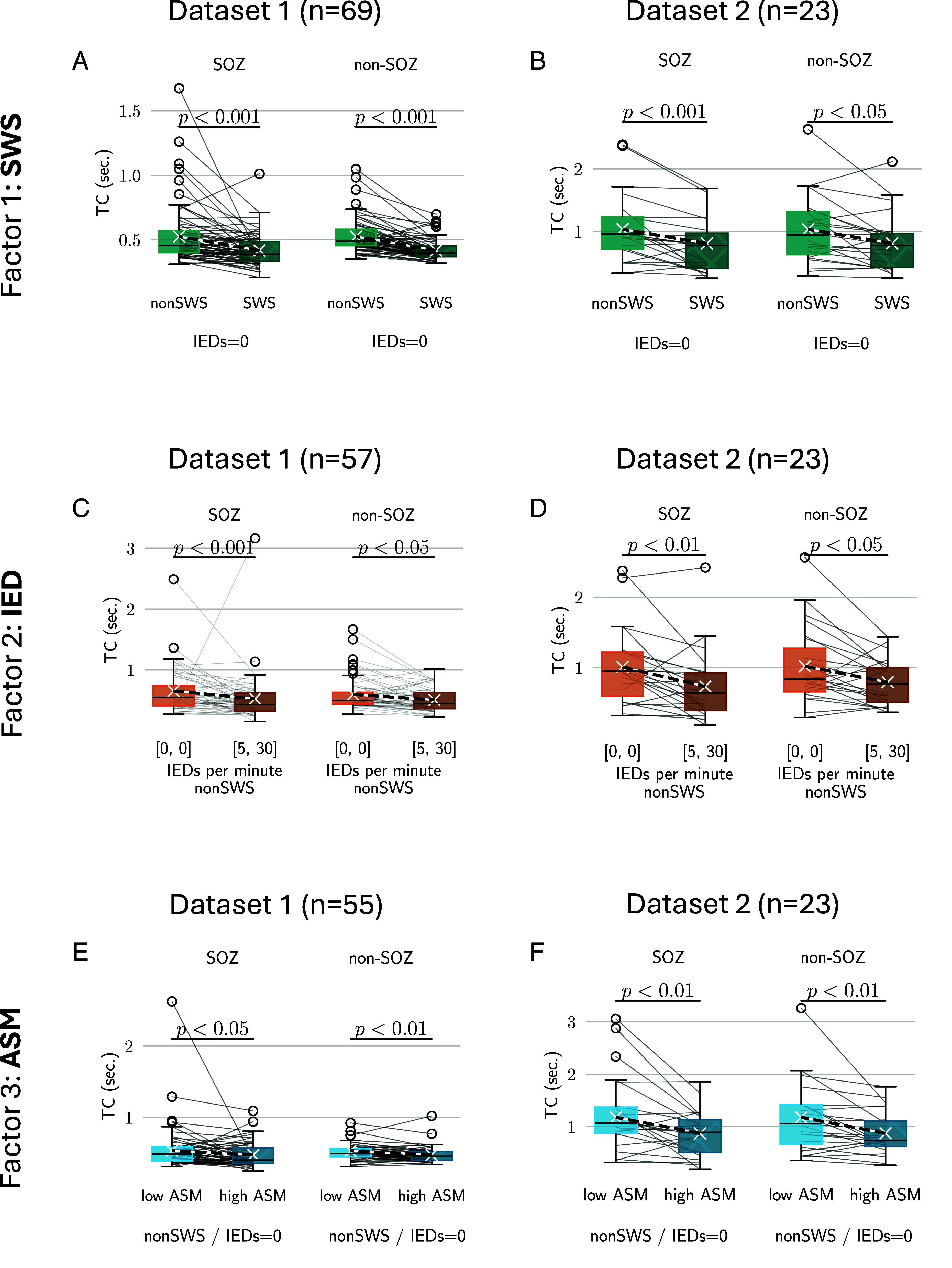
TCs decline under interictal epileptic discharges, slow wave sleep, and antiseizure medication in the seizure onset zone (SOZ) and non-SOZ. (*A*) SWS disrupts TCs both in the SOZ and the non-SOZ in dataset 1 and (*B*) dataset 2. (*C*) With more IEDs, TCs decline in both SOZ and non-SOZ in dataset 1 and (*D*) dataset 2. (*E*) TCs decline with increased ASM load in SOZ and non-SOZ dataset 1 and (*F*) dataset 2. Abbreviations: IED—interictal epileptic discharges; SWS—slow wave sleep; ASM—antiseizure medication; SOZ—seizure onset zone.

We next examined the network state and the effects of these three factors separately in the seizure onset zone (SOZ) and non-SOZ electrodes (Fig. 3). We found TCs similarly shortened in the non-SOZ and the SOZ as a function of SWSs (Fig. 3 *A* and *B*), IEDs (Fig. 3 *C* and *D*) and ASMs (Fig. 3 *E* and *F*) demonstrating the widespread network effects incurred by these factors. In particular, the effect of IEDs showed a trend of decreasing TCs with increasing IEDs (*SI Appendix*, Fig. S5). Accounting for the effect of IEDs by fitting a linear mixed effects model and investigating TCs for no IEDs revealed a trend of longer TCs in the SOZ (significant coefficient only in dataset 1, *SI Appendix*, Fig. S5*A*).

### Distance to Optimal Critical Dynamics Predicts Cognitive Impairments.

**Fig. 4.**
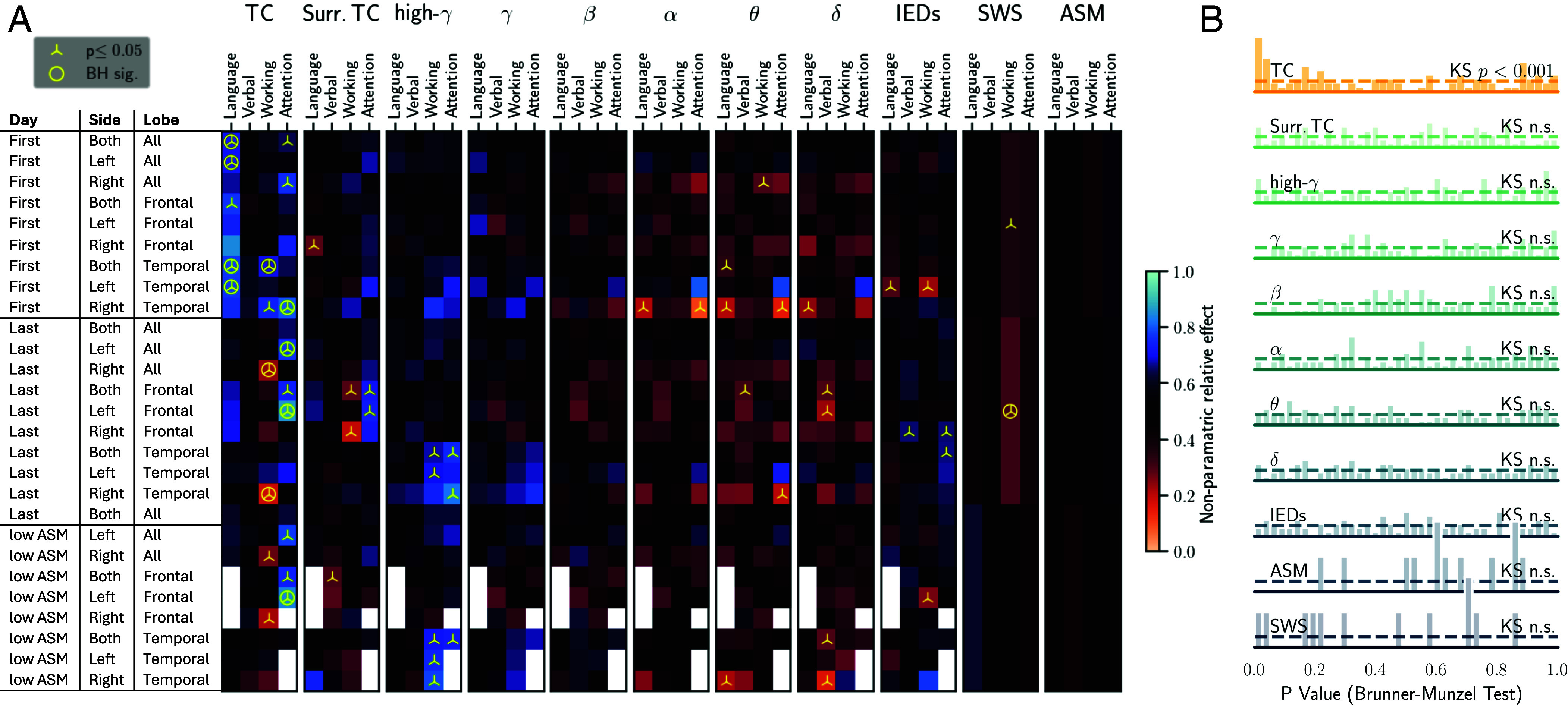
Cognitive impairment is predicted robustly only by TCs. (*A*) EEG and clinical features were compared to performance on cognitive tests, each box denotes one comparison. On the left, the table notes the temporal and spatial characteristics of sampling. We assessed EEG features during the first, last, and low ASM days, across temporal and frontal lobes, and both hemispheres. Each heatmap contains four columns in order to relate EEG features to performance in specific cognitive domains: language, verbal learning and memory, working memory, and attention. A cognitive domain was considered impaired if two or more tests within the domain were 1 SD under the healthy norm. Each heatmap column group refers to a single EEG feature examined: temporal correlations (TCs) and surrogate TCs, power spectra in the *δ* (0.5 to 4 Hz), *θ* (4 to 8 Hz), *α* (8 to 12 Hz), *β* (12 to 30 Hz), *γ* (30 to 45 Hz), and high-*γ* (55 to 95 Hz) ranges, and IEDs. In addition, SWSs and ASM load were investigated. The color within each box denotes the effect size, statistical significance is noted by a triangle at P<0.05 and by a circle after Benjamini–Hochberg correction. White boxes had insufficient sample size for analysis. (*B*) *P*-values of all comparisons.

In model and data, IEDs, ASMs and SWSs all shortened TCs. This suggests critical dynamics to be the common setpoint for optimal network function. We thus finally assessed whether distance to criticality predicted cognitive performance across all heterogeneous factors. We identified a significant relationship between TCs and cognitive impairment, with 20 out of 102 subcomparisons showing significance (P<0.05, Brunner–Munzel test; Fig. 4*A*), of which 11 retained significance after adjusting for multiple comparisons (Benjamini–Hochberg method with α=0.05). TCs were the only measure correlated with impairments in attention, language, and working memory; no other EEG features were significant after multiple comparison correction (Fig. 4*A*). Importantly, neither IEDs, ASM load, or SWSs (with the exception of one value, as a nonlocalized measure only 12 comparisons could be performed) exhibited significant predictive value for cognition (Fig. 4*A*). Additionally, the existence of lesions in this PwE cohort as detected by MRI did not correlate with any cognitive domain (P>0.05). In line with these observations, a broader analysis of the *P*-value distributions for all features revealed that only TC’s *P*-value distribution significantly deviated from a uniform distribution expected under the null hypothesis (P<0.01, one-sided Kolmogorov–Smirnov test; Fig. 4*B*). Correlations were strongest on the first day of recording, before the onset of confounding clinical factors including accumulating seizure load (12.0 ± 16.2 seizures per monitoring period), variable monitoring duration (10.9 ± 4.8 d), and medication changes (*SI Appendix*, Fig. S8).

## Discussion

**Fig. 5.**
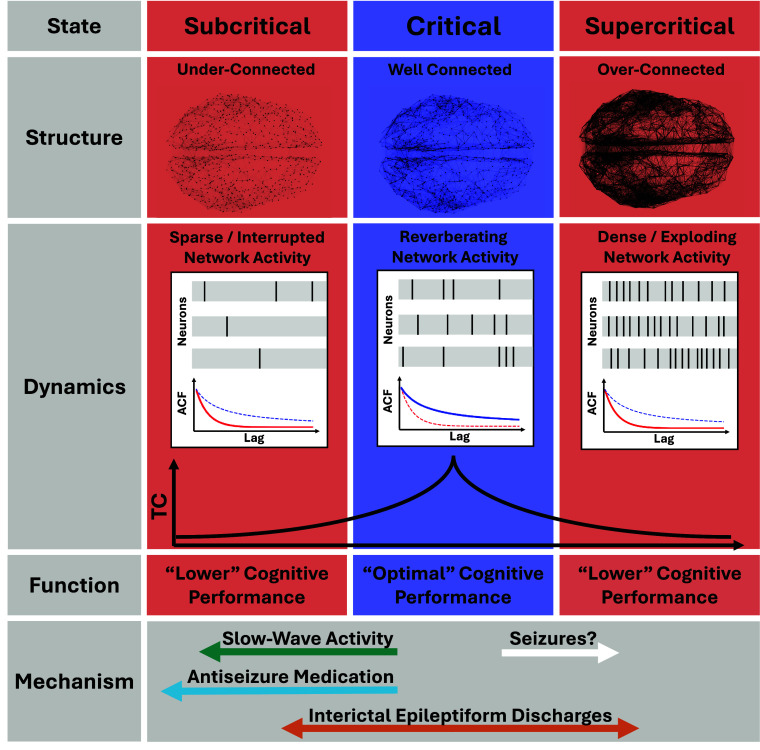
Illustration of the link between structure, dynamics, and function in the brain criticality framework and the action of perturbative mechanisms. Subcritical: Underconnected networks showing sparse and interrupted activity do not allow for the propagation of information, resulting in short TCs and lower cognitive performance. Critical: Well-connected networks with reverberating, critical dynamics exhibit maximized TCs leading to optimal cognitive performance. Supercritical: Overconnected networks exhibiting dense activity leading to a fast decay of information through chaotic interactions. In this state TCs are short and limited cognitive performance. We here presented evidence that low TCs predict lower cognitive performance and that three distinct mechanisms, slow-wave activity (SWSs), ASMs, and interictal epileptiform discharges (IEDs) push the systems away from optimal critical dynamics.

We provide evidence that TCs, a hallmark of critical dynamics, predict cognitive performance profiles across multiple domains. We show that factors known to impair cognition, including IEDs, ASMs, and SWS, all act directly to perturb critical dynamics and thus cognition. Our work suggests critical dynamics to be the setpoint to determine optimal network function, thereby providing a unifying framework for the heterogeneous mechanisms impacting cognition in conditions like epilepsy (Fig. 5).

Cortical networks have long been hypothesized to operate in the vicinity of a critical state where they can benefit from optimized function by balancing efficient information processing, memory, and flexibility. While previous work has established connections between critical dynamics and specific cognitive functions, studies have been limited by the constraints of noninvasive recording methods (33–35, 37–39, 42, 43, 56–59). These include short EEG recording durations that may be limited in adequately capturing the variability of cortical network states, by coarse spatial brain coverage, and by artifacts and signal filtering. Importantly, high-γ power activity, the closest proxy to neuron population firing (47, 48), is typically filtered out by the scalp in surface EEG and is even less resolved in slowly fluctuating signals like the fMRI BOLD signal. Additionally, many studies have focused on single cognitive tasks rather than comprehensive assessment of cognitive profiles. We addressed these limitations by analyzing iEEG recordings characterized by high spatial resolution and long recording duration, allowing us to capture high-*γ* power fluctuations that closely capture local spike rate variations (47–49, 60, 61). Thus, we can evaluate TCs derived from high-*γ* signal, reflecting information dissipation indicating the critical state (49, 58–61).

Our hypothesis based on criticality theory and results from our computational model was that disrupted TCs are a signature of cognitive impairment. Our empirical results indicate that TCs are robustly shorter for patients with cognitive impairments, with the strongest relationships observed in language and attention domains during the first full day of EEG recording. The strongest effects on day one likely reflect minimal interference from hospitalization-related factors that could alter patients’ baseline states from cognitive testing, including medication changes (affecting 82% of patients), accumulating seizure load, and variable duration of monitoring (*SI Appendix*, Fig. S8). Nonetheless, significant findings were also demonstrated on the last recording days and lowest ASM dose days. To note, we employed multiple approaches to verify the robustness of our findings. Analysis of frequency band powers, including high-γ power used to derive TCs, showed no significant relationship with cognitive outcomes, indicating that our results are not explained by artifactual or general signal properties. IED rates similarly showed no correlation with cognitive performance, indicating that results are not simply reflecting disease severity. Statistical comparisons to null distributions, multiple testing corrections, and controls for SWS and ASM dosage further validate our findings.

Our results also shed light on the underlying mechanisms of cognitive impairments in conditions like epilepsy by examining how three cognitive-related factors—SWS, IED activity and ASM load—disrupt TCs and critical dynamics.

Examining SWS, our model’s predicted TC disruption was validated across two independent datasets. This aligns with theories that during sleep and sleep-like periods, the brain’s ability to effectively integrate information across cortical areas and time is reduced (62), while extended wakefulness leads to intermittent local sleep activity that impairs cognition and disrupts the optimal critical state (29, 30, 63). In PwE, this is an especially pertinent consideration as epilepsy disturbs sleep, influencing sleep structure, architecture, continuity, and oscillations (64).

IEDs were simulated by our model as probabilistic activations of local neuronal populations. Our model predicted that IEDs would disrupt TCs, and this was confirmed in both our datasets, with a dose-dependent relationship between IED frequency and TC reduction. This finding suggests that increased disease activity is directly related to a shift away from the critical state and may provide a mechanistic perspective to the previously reported “transient cognitive impairment” phenomenon, which describes impaired cognitive performance due to concurrent epileptic activity (65, 66). It may also potentially shed light on neuronal mechanisms underlying impaired cognition associated with IED activity reported in neurodegenerative disorders including Alzheimer’s disease (9). Although we demonstrate a drift away from criticality with increased IEDs, we cannot draw direct conclusions if the deviation is toward sub- or supercritical dynamics. One prior study suggested that IEDs themselves are a display of supercritical dynamics (67), however additional work is needed to resolve this open question.

ASM effects, both in our model and empirical data showed increased ASM doses shortened TCs, extending findings from prior works (27, 28). ASM treatment is recognized to have a dose-dependent detrimental effect on cognition in PwE (68). One hypothesis is that the reduction of neuronal excitability through ASMs (69, 70) can lead to decreased effective connectivity between the neurons and thus a transition toward subcritical dynamics and consequentially impaired cognition (71). In this study, ASM dosage in itself did not relate to cognition, possibly since all patients were highly drug-resistant.

Findings of the effects of factors were consistent across both seizure and nonseizure onset tissue, suggesting that our findings may not only be an epilepsy-specific effect. In dataset 1, we found a stronger decline of TCs with increasing IEDs in the SOZ compared to the non-SOZ. Further, correcting for IEDs in the SOZ revealed slightly larger TCs than in the non-SOZ. This finding may support the hypothesis that the SOZ is closer to criticality and thus is more prone to drifting into a supercritical state, a phenomenon which is also linked to seizure initiation (72, 73). To note, in dataset 2, we could not establish this finding, possibly due to a smaller sample size, similarly to a prior study (74).

Our study has several limitations. First, dataset 1 was limited to 5-min recordings each hour rather than continuous monitoring. However, PwE were monitored for long durations, and we had a large sample size of over 100 patients. Second, for IED and SWS segment detection, we relied on validated algorithms rather than manual annotation. Furthermore, it was not possible to perform sleep scoring according to polysomnographic standards as PwE did not have EMG or scalp EEG recordings. Third, while our results demonstrate a clear relationship between cognitive profiles and TC duration, we acknowledge that effects on cognition are multifactorial and dynamic. Cognitive assessment was performed as part of presurgical workup according to best practice clinical standards but necessarily reflected a single timepoint and was not concurrent with iEEG recordings—a common limitation in studies linking cognition to brain function (58). This timing mismatch could introduce bias given the nature of cognitive impairment in epilepsy and variations in ASM dosage, though we mitigated this through the use of a comprehensive test battery. Fourth, as intracranial electrodes were predominantly implanted in temporal and frontal regions, the interpretation of cognitive domains affected should be done accordingly—potentially affecting the predominance of findings within the language and attention domains.

Collectively, our results from model simulations and iEEG recordings show that TCs decline under perturbative mechanisms—SWS, IEDs, and ASM—and that shorter TCs predict cognitive impairment in PwE. More generally, our results demonstrate how dynamical factors may distinctly influence the brain’s distance from a critical state and thereby cognition (Fig. 5). While these findings suggest potential therapeutic targets, they also highlight a fundamental challenge in epilepsy: interventions aimed at optimizing critical dynamics for cognitive function must carefully consider the risk of increasing seizure susceptibility, as proximity to criticality may also lower the threshold for seizure generation (72, 75). In other words, seizures may be the price for a brain being tuned to optimal computation. In conclusion, our work suggests critical dynamics to be the setpoint to measure optimal network function, thereby providing a unifying framework for the heterogeneous mechanisms impacting cognition in conditions like epilepsy.

## Supplementary Material

Appendix 01 (PDF)

## Data Availability

Code for the model has been deposited in (https://gitlab.com/computational-neurologie/stc_model_2024). Code for the analysis is available under https://gitlab.com/computational-neurologie/sde_n_cog for dataset 1 and https://gitlab.com/computational-neurologie/ieegCD for dataset 2. Dataset 2 is publicly available within the Epilepsiae database (49).
