# Supplementary material for: Critical dynamics predicts cognitive performance and provides a common framework for heterogeneous mechanisms impacting cognition
Source: Proc Natl Acad Sci U S A. 2025 Apr 3;122(14):e2417117122. doi: 10.1073/pnas.2417117122 (PMC12002245; doi:10.1073/pnas.2417117122)
Supplement: Supplementary file 1 — Appendix 01 (PDF) [file pnas.2417117122.sapp.pdf]

Supplementary Material

Supplementary Material 1: Scaling of the connection strength and the largest absolute eigenvalue

To understand how we scale the connection strength in our model simulation one can investigate the properties of eigenvalues and matrix multiplication. Let $\boldsymbol{W}$ be our connectivity matrix, u_i an eigenvector and μ_i an eigenvalue, i.e.,

$$\boldsymbol{W}u_i = \mu_i u_i\,.$$

If we scale $\boldsymbol{W}$ by a constant factor k we get a new scaled matrix $\boldsymbol{W}'$

$$k\boldsymbol{W}u_i = k\mu_i u_i\,,$$
$$(k\boldsymbol{W})u_i = (k\mu_i)u_i\,,$$
$$\boldsymbol{W}'u_i = \mu_i' u_i.$$

This new matrix has the same eigenvectors but the eigenvalues are also scaled by the factor k. Now we will do this for the largest absolute eigenvalue $\lambda > 0$ (with the eigenvector u_λ) and want to arrive at a new scaled matrix $\boldsymbol{W}'$ with a largest absolute eigenvalue $\lambda' = 1$. To achieve this, we can just set $k = \frac{1}{\lambda}$

$$\boldsymbol{W}' = \frac{1}{\lambda}\boldsymbol{W}u_\lambda = \frac{1}{\lambda}\lambda u_\lambda = u_\lambda.$$

Starting from this matrix $\boldsymbol{W}'$ we can get a matrix with an arbitrary largest absolute eigenvalue by scaling $\boldsymbol{W}'$ with the desired largest absolute eigenvalue.

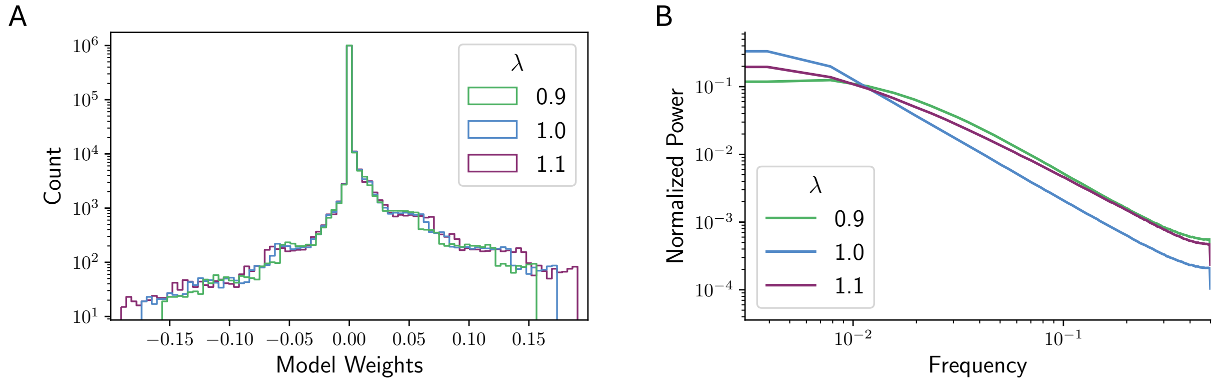

Figure S1: A, Model weight distribution for three different networks with $\lambda \in \{0.9, 1.0, 1.1\}$. B, Power spectral density of the model's activity for the same λ does not show any confined oscillatory.

Supplementary Material 2: Model activity and data high-γ power for SWS, IEDs, and ASMs

The model discussed in the main manuscript is a branching-type model, which has a phase transition between vanishing and exploding activity (Figure 1 D). This simple-to-track model allows the study of different observables, but other types of phase transition are also conceivable to occur in cortical networks, e.g., between asynchronous and synchronous firing [1]. For example, our model shows a decrease in activity under SWS which is reflected in a decrease of the high-γ power in the data (Figure S2 A-C). However, for IEDs the model exhibits an increase in activity while the data is less conclusive, and only an increase in high-γ power in dataset 1 could be observed (Figure S2 D-F). For ASMs the model shows a decrease in the activity while this trend could not be observed in the data at all (Figure S2 G-I). This indicates that power alone is not a good marker for the critical transition in particular when the type of phase transition is not known. However, TCs are a more robust marker for the phase transition, independent of the type of the phase transition and results between the model and the data are robust (Figure 1, 2 in the main manuscript).

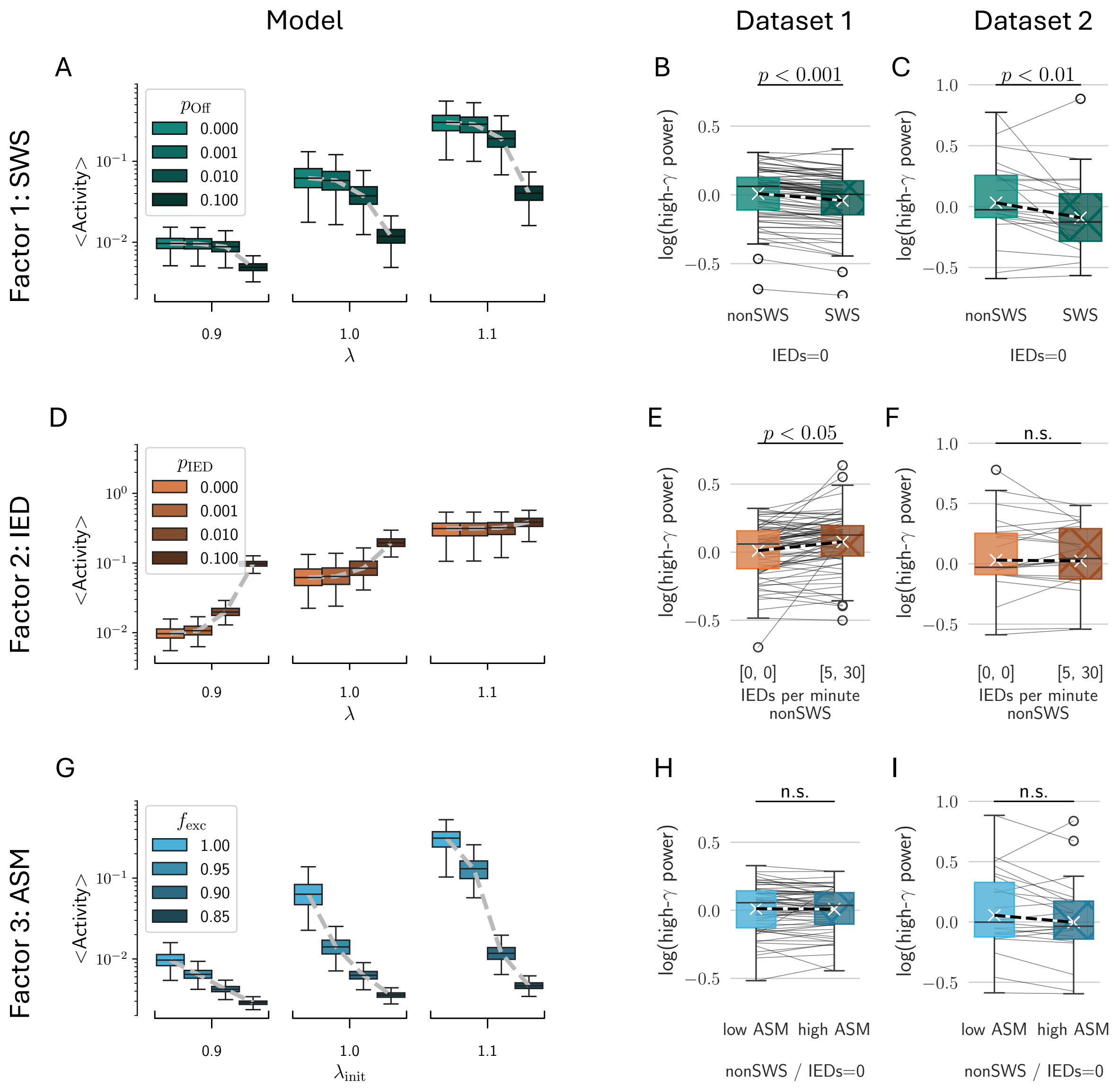

Figure S2: Model activity and high-γ power with respect to SWS, IEDs, and ASMs. A, The model activity declines with more off-periods in the model. Similarly, the high-γ power is reduced under SWS compared to nonSWS (B, dataset 1and C, dataset 2). D, More IEDs in the model lead to more activity. E, In dataset 1 an increase of the high-γ power can be observed with more IEDs but not in dataset 2 (F). G, The model shows a decrease of the activity with increasing ASM effects. No such change can be found in the high-γ power in dataset 1 or I dataset 2 (H, I). Whiskers extend to 1.5 times the interquartile range. Mean is shown as white crosses for the data and single patient data is shown with thin black lines. Significance was tested with Wilcoxon signed-rank test.

Supplementary Material 3: Hurst Exponents align with TCs but are associated weaker with SWS, IEDs and ASM

Detrended Fluctuation Analysis (DFA) is a widely-used method to characterize long-range temporal correlations in time series data, particularly in non-stationary signals [2,3]. DFA yields Hurst exponents, which quantify the self-similarity of fluctuations across different time scales, with values greater than 0.5 indicating long-range temporal correlations. Hurst exponents approaching 1 have consequently been associated with critical dynamics. We also performed DFA on our model and empirical data to validate our findings based on autocorrelation analysis. We found that Hurst exponents peaked near criticality in our model ($\lambda \approx 1$) and showed significant correlation with the temporal correlations (TCs) derived from autocorrelation functions (Spearman $\rho = 0.6$; $p <$machine precision). In the empirical data, specifically in dataset 1 where recordings spanning multiple days were available, we observed significant within-patient correlations between Hurst exponents and TCs in line with model predictions (Spearman $\rho = 0.4 \pm 0.4;\ p < 0.001$). While we found increased Hurst exponents during SWS and higher IED rates in dataset 1 (Figure S3 E, G), these changes were also accompanied by increased signal-to-noise ratios (Figure S6 A, C) and were not robust in dataset 2 (Figure S3 F, H). However, the relationship between ASM load and temporal organization was consistently reflected in both Hurst exponents and TCs, with lower values during high ASM days in both datasets (Figure S3 I, J). Importantly, between different ASM loads, we did not find changes in the signal-to-noise ratio estimations (Figure S6 E, F).

These findings thus suggest that, while DFA provides valuable complementary information about temporal correlations, TC estimation via autocorrelation functions may be more robust against amplitude changes in the signal.

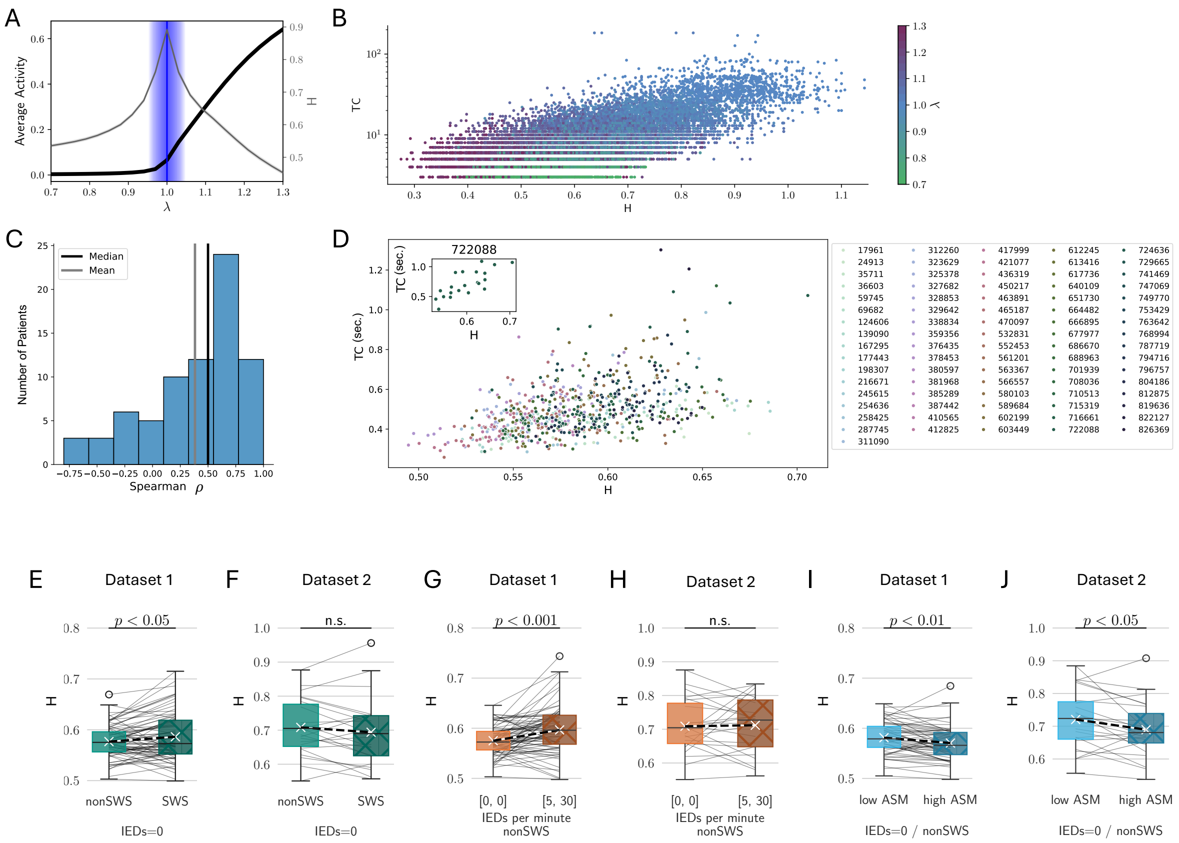

Figure S3: Hurst exponents in the model and data. Detrended fluctuation analysis was performed in the model and data as a second estimate for proximity to criticality and temporal organization in the signal. A, In the model, Hurst exponents (H) peak at criticality, i.e., $\lambda \approx 1$. B, In the model, Hurst exponents were significantly correlated with TCs for varying λ (Spearman $\rho=0.6$; p< machine precision). C, Dataset 1 had more than two days of data so that we extracted day-wise TCs and Hurst exponents from the high-γ power fluctuations in nonSWS segments. Across patients, the within-patient correlation between Hurst exponents and TCs was significantly positive (Spearman $\rho=0.4\pm0.4$; $p<0.001$ Wilcoxon signed-rank test). D, Correlation between H and TC with one data point per day. The patient with the most data points is shown as inset. E, In dataset 1, we found slightly higher Hurst exponents in SWS than in nonSWS but not in dataset 2 (F). G, In dataset 1, we found an increase in Hurst exponents with more IEDs. H, However, this change was not robust in dataset 2. I, In dataset 1 and dataset 2 (J), we found that Hurst exponents were longer during low ASM days, aligning with our results of TCs (Figure 2 E in the main manuscript). Whiskers extend to 1.5 times the interquartile range. Means are shown as white crosses for the data, and single-patient data is shown with thin black lines. Significance was established with the Wilcoxon signed-rank test.

Supplementary Material 4: TCs in the α – band

To examine whether temporal correlations (TCs) in different frequency bands show similar behaviors, we analyzed TCs in the α-band (8-12 Hz) alongside our primary high-γ analysis (Supplementary Figure S4). While α -band TCs partially aligned with high-γ-TCs and the model, in particular showing similar trends regarding ASM effects (Figure S4 H, I), we observed notable differences in other conditions. α-TCs showed no significant association with SWS (Figure S4 B, C), contrasting with the decreased TCs observed in the high-γ-band. α-TCs demonstrated an opposite relationship with IEDs compared to high-γ-TCs, showing increased rather than decreased TCs with higher IED rates (Figure S4 E, F). This increase in α-TCs during periods of high IED activity could reflect the influence of IEDs, which are characteristic 20-200ms high-amplitude transients which are thus non-stationarities corresponding to the 5-50 Hz frequency range. Therefore, IEDs could potentially create artificial long temporal correlations in lower frequency bands such as α.

Collectively, these findings support our focus on high-γ activity, which provides the most direct window into underlying neuronal dynamics [4–8]. Nevertheless, α -band analysis may prove valuable in settings where high- γ activity cannot be assessed, such as in non-invasive EEG recordings.

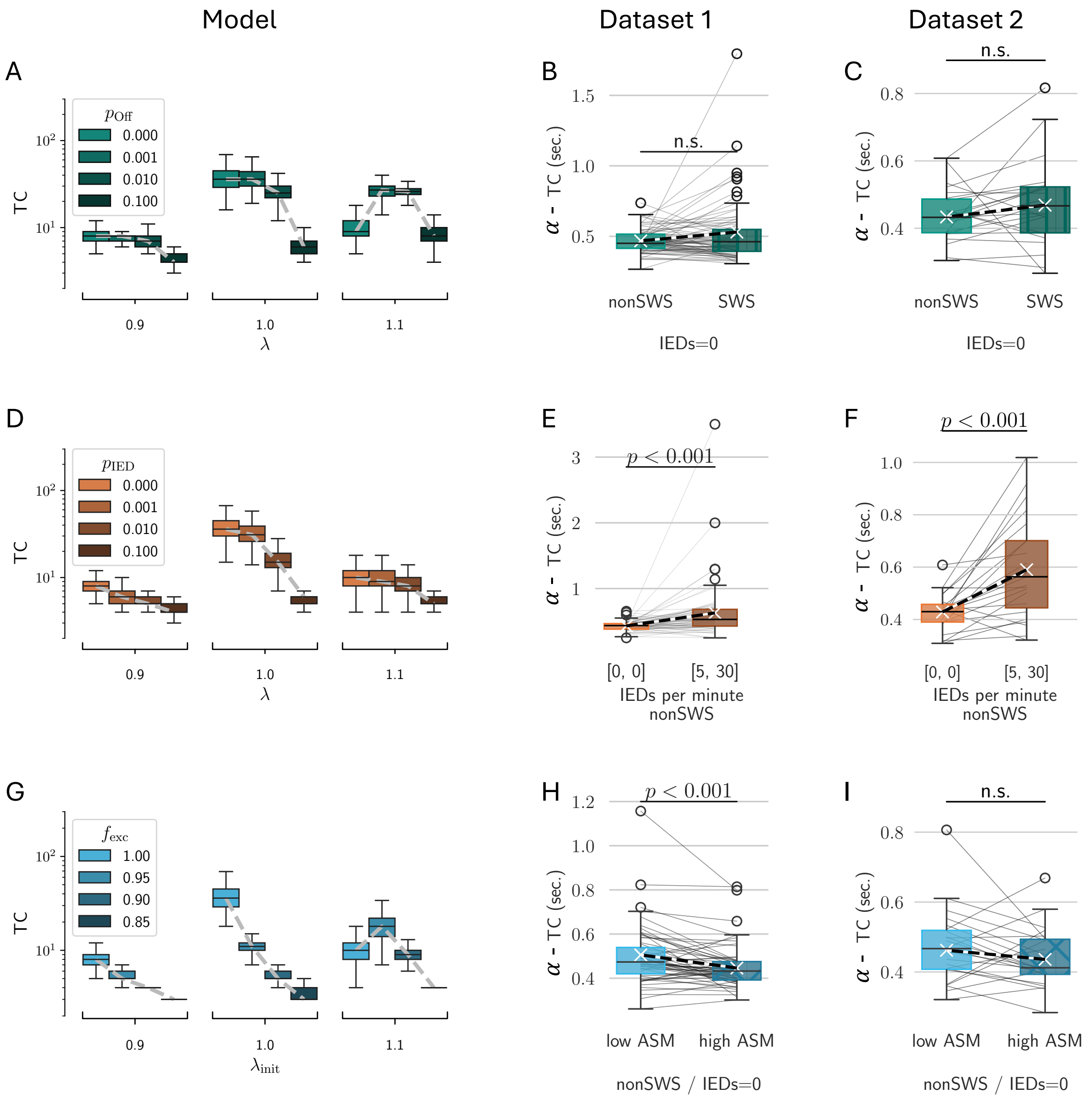

Figure S4: α-band TCs with respect to SWS, IEDs, and ASM. A, For SWS the model predicts a decline of TCs. B, However, no significant change of α – band TCs was found in dataset 1 or dataset 2 (C). D, For IEDs the model predicts a decrease of TCs. E, However, the data shows an opposite trend of an increase of α – band TCs with more IEDs in dataset 1 and dataset 2 (F). G, For ASMs the model predicts a decline of TCs. H, This trend can be found for α – band TCs in dataset 1, too, but does not reach significance in dataset 2 (I).

Supplementary Material 5: Linear mixed effects model for increasing IED effect on TCs

To analyze the effect of increasing IEDs on TCs, we compared TCs between the following IED per minute count bins: no IEDs, (0-1], (1-5], and (5-30] IEDs per minute. The influence over all IED bins on TCs was then evaluated using a linear mixed effects model: dependent variable: TC; independent variables: ordered IEDs, SOZ or non SOZ, interaction of term SOZ and IEDs; random effect: patient ID.

In both datasets, we observed that TCs were progressively shortened under increasing numbers of IEDs in the SOZ and non-SOZ (Figure 3 B, E). For this analysis, to allow comparability across patients, we included only PwE who had at least 50 segments in each IED bin for at least one channel, excluding SWS segments. This is a trade-off between the accuracy of the autocorrelation estimation and the sample size with respect to channels and patients. In dataset 1 (comprised of 50 eligible PwE), we found negative linear and quadratic IED coefficients (linear: -0.13 [-0.20, -0.07] [Wald confidence intervals], t=-3.9; quadratic: -0.069 [-0.0136, -0.02], t=-2.0). The former shows that with increasing IEDs TCs were progressively shorter and the latter indicating that this decline was decelerating for higher IED counts. Additionally, the model revealed that non-SOZ areas, compared to SOZ areas, had smaller baseline TCs (-0.052 [-0.997, -0.02], t=-2.1). This effect was less marked in dataset 2, where all 23 patients were eligible. Here, only the linear decrease in TCs with increasing IED bin was robust (-0.24 [-0.35, -0.13], t=-4.1), with no robust effects discerning the SOZ from the non-SOZ. The prediction of the models is illustrated in Supplementary S1. Together these observations thus indicate a more rapid decline of TCs as a function of IEDs in the SOZ and overall higher baseline TCs in the SOZ.

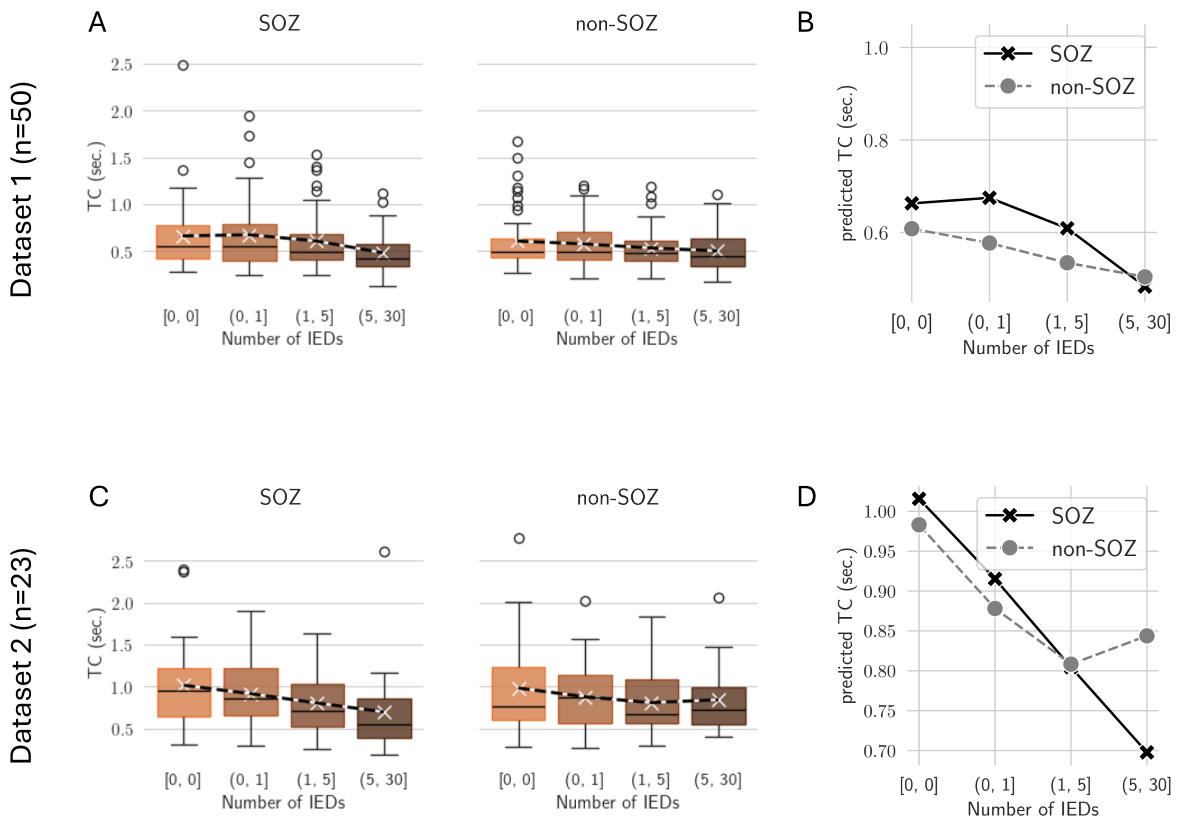

Figure S5: With more IEDs TCs progressively decline irrespective of seizure onset zone (SOZ) or non-SOZ. A, For dataset 1, 50 patients had enough data in all 4 IED bins. B, The predictions of the linear mixed effects model illustrate the progressive decline of TCs with more IEDs and show that the SOZ might have longer TCs than the non-SOZ. C, A similar decline of TCs with more IEDs can be observed in dataset 2 for the SOZ and non-SOZ. D, The linear mixed effects model predictions show a similar trend but the difference between SOZ and non-SOZ is less pronounced.

Supplementary Material 6: The estimated signal-to-noise ratio cannot explain the trends of TCs

To assess whether changes in signal-to-noise ratio (SNR) could influence TCs, we employed the FOOOF (Fitting Oscillations & One Over F) algorithm to estimate SNR [9]. FOOOF models neural power spectra as a combination of an aperiodic background component and periodic oscillations, allowing us to use its high-γ-component normalized by the aperiodic background as a proxy for SNR.

In dataset 1, we observed significant increases in the estimated SNR during SWS and in segments with higher IED rates (Figure S6 A, C). However, these changes were in the opposite direction of TC changes, and the relationship was not robust in dataset 2 (Figure S6 B, D). Importantly, we found no association between the estimated SNR and ASM load in either dataset (Figure S6 E, F). To examine this relationship more precisely, we leveraged the extended recording duration in dataset 1 to directly correlate TCs with the estimated SNR (Figure S6 G, H), finding no significant correlation on the population level (Spearman $\rho = 0.1 \pm 0.5;\ \ p = 0.2$ Wilcoxon signed-rank test). Together, these findings demonstrate that TC estimates reflect genuine temporal organization in neural activity rather than being artifacts of changes in signal power or SNR.

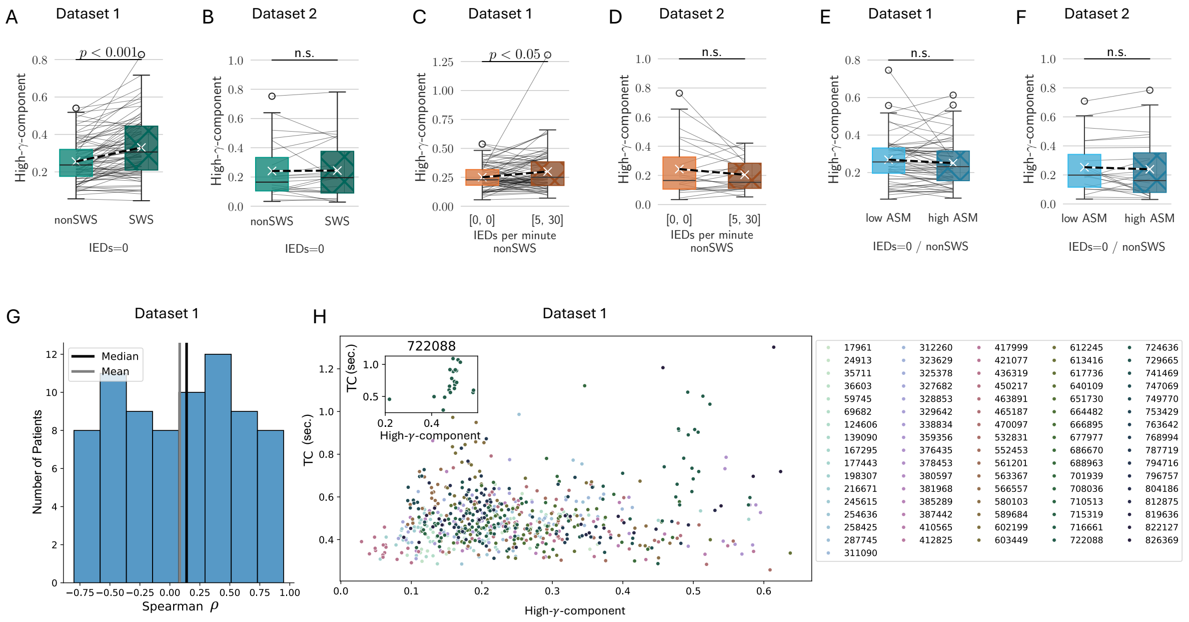

Figure S6: Signal-to-noise ratios are estimated as the high-γ-component of the oscillations (normalized by the aperiodic background) extracted via the FOOOF (Fitting Oscillations & One Over F) algorithm. A, Dataset 1 showed increased high-γ-component during SWS, but dataset 2 did not (B). C, Dataset 1 showed a higher high-γ-component with more IEDs which was not seen in dataset 2 (D). No changes were observed with respect to ASM load for dataset 1 (E) or dataset 2 (F). G, Dataset 1 had more than two days available, which allowed us to estimate the correlation between TCs and the high-γ-component. The distribution of Spearman ρ was centered around zero. H, No significant correlation between TCs and the high-γ-component were observed. The inset shows the patient with the most available days.

Supplementary Material 7: Cognitive Testing

All patients underwent a test battery including 14 cognitive tests, as part of their clinical evaluation. Test scores were normalized according to established norms provided in the test manuals, based on validated samples of healthy individuals, and raw scores were converted to percentiles accordingly. Impaired individuals had a score of at least 1 SD below from the norm in more than one test within a domain, with each domain represented by 3-4 tests. In total, there were 67 missing values (of 1134 data points, average of 0.8±1.7 data points per patient). Fifty-four (66%) patients had impaired language, 37 (45%) impaired working memory, 32 (39%) impaired verbal memory, and 17 (20%) patients impaired attention. These missing values were iteratively imputed using *scikit-learn*'s iterative imputer[10]. Cognitive tests and their distributions are summarized in Figure S7. Verbal learning and memory were evaluated using a word list learning test, the German version of the Rey Auditory Verbal Learning Test ("Verbaler Learn- und Merkfähigkeitstest")[11]. Parameters assessed included immediate memory, reproduction of the last learning trial, total learning as well as delayed recall. Language was evaluated using semantic and phonetic word fluency tests with and without category switching ("Regensburger Wortflüssigkeitstest")[12]. Working/short term memory was assessed by two block tapping tests and Mottier tests; and attention was assessed using the computerized Test battery for Attentional Performance (TAP; "Testbatterie für Aufmerksamkeitsprüfung") with the subtests Alertness and Go/No-Go[13–15].

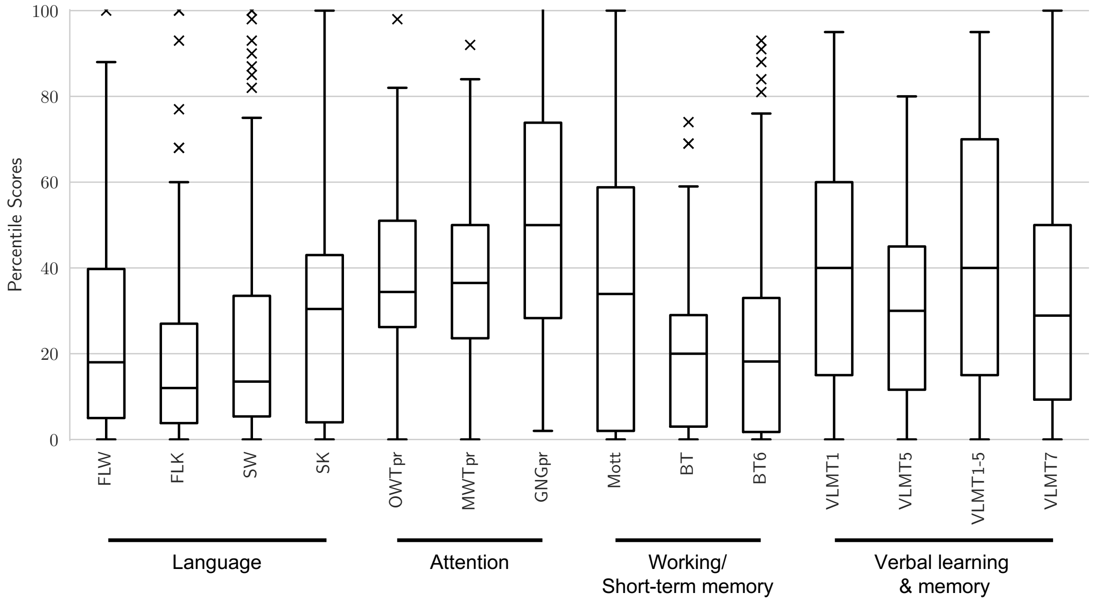

Figure S7: All cognitive tests and their distributions as performed during the clinical assessment of patients. Abbreviations: FLK – phonetic word fluency, FLW - phonetic category switching, SK – semantic word fluency, SW - semantic category switching; attention, Testbatterie für Aufmerksamkeitsprüfung (TAP) Attention subtest: OWTpr performed without auditory warning cue, MWTpr- with auditory warning cue , GNGpr- Go/No-Go subtest. Working/short term memory- BT, BT-6- Block Tapping, Mott- Mottier test. Verbal learning and memory: VLMT - Verbaler Lern- und Merkfähigkeitstest; VLMT1 - immediate memory; VLMT5 - reproduction of the last learning trial; VLMT1-5 - total learning; VLMT7 - delayed recall.

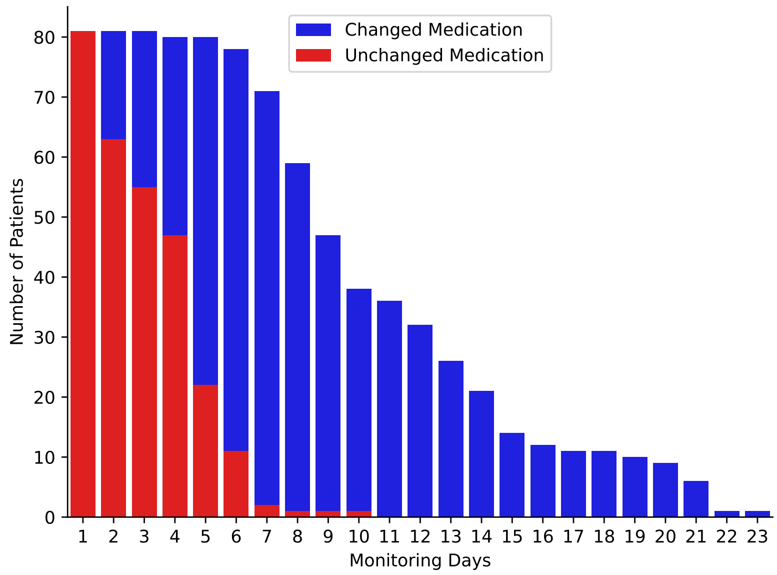

Figure S8: Medication changes of all patients across video-EEG monitoring days. For each day of monitoring on the x-axis, the number of patients that had unchanged medication doses is shown by the red column, and the number of patients that underwent medication tapering is shown in blue.

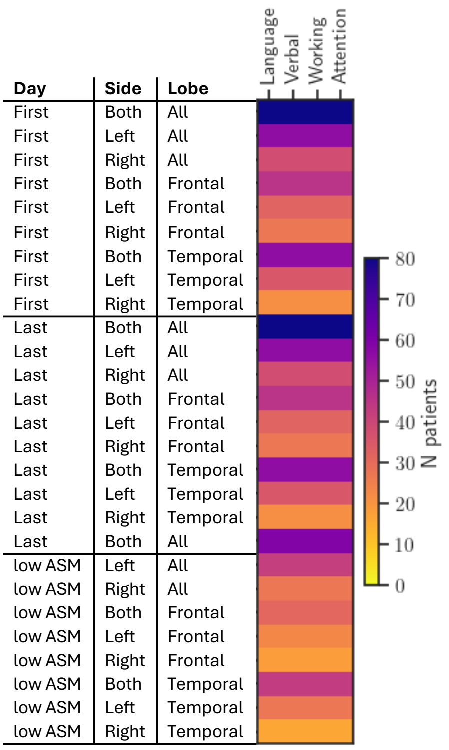

Figure S9: Total number of patients for each observation day.

References:

1. Li, J. & Shew, W. L. Tuning network dynamics from criticality to an asynchronous state. *PLoS Comput Biol* **16**, e1008268 (2020).
2. Hardstone, R. *et al.* Detrended Fluctuation Analysis: A Scale-Free View on Neuronal Oscillations. *Front. Physio.* **3**, (2012).
3. Linkenkaer-Hansen, K., Nikouline, V. V., Palva, J. M. & Ilmoniemi, R. J. Long-Range Temporal Correlations and Scaling Behavior in Human Brain Oscillations. *J. Neurosci.* **21**, 1370–1377 (2001).
4. Nir, Y. *et al.* Coupling between Neuronal Firing Rate, Gamma LFP, and BOLD fMRI Is Related to Interneuronal Correlations. *Current Biology* **17**, 1275–1285 (2007).
5. Manning, J. R., Jacobs, J., Fried, I. & Kahana, M. J. Broadband Shifts in Local Field Potential Power Spectra Are Correlated with Single-Neuron Spiking in Humans. *Journal of Neuroscience* **29**, 13613–13620 (2009).
6. Miller, K. J. Broadband Spectral Change: Evidence for a Macroscale Correlate of Population Firing Rate? *Journal of Neuroscience* **30**, 6477–6479 (2010).
7. Whittingstall, K. & Logothetis, N. K. Frequency-Band Coupling in Surface EEG Reflects Spiking Activity in Monkey Visual Cortex. *Neuron* **64**, 281–289 (2009).
8. Ray, S. & Maunsell, J. H. R. Different Origins of Gamma Rhythm and High-Gamma Activity in Macaque Visual Cortex. *PLoS Biol* **9**, e1000610 (2011).
9. Donoghue, T. *et al.* Parameterizing neural power spectra into periodic and aperiodic components. *Nat Neurosci* **23**, 1655–1665 (2020).
10. Pedregosa, F. *et al.* Scikit-learn: Machine Learning in Python. *Journal of Machine Learning Research* **12**, 2825–2830 (2011).

11. Helmstaedter, C. & Durwen, H. F. [The Verbal Learning and Retention Test. A useful and differentiated tool in evaluating verbal memory performance]. *Schweiz Arch Neurol Psychiatr (1985)* **141**, 21–30 (1990).

12. Aschenbrenner, S., Tucha, O. & Lange, K. W. *Regensburger Wortflüssigkeits-Test: RWT*. (Hogrefe, Verlag für Psychologie, 2000).

13. Zimmermann, P. & Fimm, B. *Testbatterie Zur Aufmerksamkeitsprüfung:(TAP)*. (Psytest, 1992).

14. Welte, V. DER MOTTIER-TEST, EIN PRUEFMITTEL FUER DIE LAUTDIFFERENZIERUNGS FAEHIGKEIT UND DIE AUDITIVE MERKFAEHIGKEIT. (1981).

15. Kessels, R. P., Van Zandvoort, M. J., Postma, A., Kappelle, L. J. & De Haan, E. H. The Corsi block-tapping task: standardization and normative data. *Applied neuropsychology* **7**, 252–258 (2000).
